# Durability as an independent parameter of endurance performance in cycling

**DOI:** 10.1186/s13102-025-01238-8

**Published:** 2025-07-10

**Authors:** Artur Barsumyan, Christian Soost, Raman Shyla, Jan Adriaan Graw, Christopher Bliemel, Rene Burchard

**Affiliations:** 1 Faculty of Medicine, Philipps-University of Marburg, Marburg, Germany; 2 Faculty III: Statistic and Econometrics, University of Siegen, Siegen, Germany; 3 Department of Anaesthesiology and Intensive Care Medicine, Ulm University Hospital, Ulm, Germany; 4 Department of Orthopaedics and Traumatology, University Hospital of Giessen and Marburg, Marburg, Germany; 5 c/o Sports Medicine and Joint Centre, Department of Orthopaedics and Trauma Surgery, Philipps- University of Marburg, Lahn-Dill-Kliniken, Rotebergstr. 2, 35683 Dillenburg, Germany

**Keywords:** Durability, Fatigue resistance, Cycling, VO_2_ max, Performance test

## Abstract

**Background:**

Recent advances in sport physiology have shown, that higher fatigue resistance predicts outstanding performance in endurance sport. However, so far there is no clear consensus on how to test durability in the field or in a laboratory. Protocols of the few existing studies are only suitable for professional male cyclists while most coaches work primarily with amateur athletes. Moreover, it is currently unclear whether durability is dependent on traditional parameters of endurance performance, such as functional threshold power (FTP) or maximal oxygen uptake (VO_2_ max).

**Methods:**

20 well trained amateur road cyclist completed a home-based test on two occasions. The first time, after a standardized warm-up, a 5-minute and a 20-minute cycling test were carried out. The second test was preceded by a fatigue protocol which, after the warm-up, consisted of cycling at 80% of their initial 20-minutes power under fresh condition until 1000 kJ of work was completed, followed by 5-minutes and 20-minutes all-out tests.

**Results:**

The performance significantly decreased with 10,1 ± 6,5% in the 20-minutes test and with 10,8 ± 7,8% in 5-minutes in fatigue state in compare with fresh state. No significant correlations were found between better durability and VO_2_ max or relative FTP.

**Conclusion:**

We showed that durability is a parameter independent of traditional physiological measures of cycling performance. Looking at durability then working backwards can help identify what coaches need to work on in so many areas that are important to all aspects of racing in cycling sport.

## Introduction

Recent research into performance factors in professional cycling has shown that maintaining a higher power output with increasing workload is a key determinant for success in professional cycling [1]. The demands of elite road cyclists often include more than 4 h (h) total race duration with a distance of more than 160 km and an altitude difference of more than 2000 m, which leads to a shift in the relationship between performance and endurance [2, 3]. The ability to minimize this shift in physiological capacity has been named “durability” [4]. Therefore, durability might serve as the “fourth dimension” alongside the traditional three parameters of endurance performance: maximal oxygen uptake (VO_2_ max), sustainable percentage of VO_2_ max, and exercise economy [5].

So far, most studies have predicted success in professional cycling based on critical power, i.e. a cyclist’s performance profile [6, 7]. However, in this case it is still questionable why two athletes with the same profile do not achieve the same performance. A recent publication suggests that the smaller drop in performance over a duration of 10 s to 20 min could be used to differentiate between successful and less successful athletes [7].

Several fatigue protocols have been proposed to assess durability. Valenzuela et al. [6] developed one of the first field-based tests using a sample of twelve professional male cyclists. The study by Bejder et al. [8] applied a high-intensity fatigue protocol in ten professional cyclists. Leo et al. [9] examined the accumulation of work using two distinct protocols in nine elite or international-level road cyclists.

Several field-based fatigue protocols have been developed to assess durability in trained cyclists. Valenzuela et al. [6] proposed a protocol in which cyclists performed a 20-minute time trial under two conditions: (1) in a rested state and (2) following a prolonged submaximal ride (~ 4 h, 40 kJ/kg). Leo et al. [9] used two different 2.5-hour fatigue protocols (moderate and high intensity) followed by two 12-minute field tests, one conducted in a fresh state and one in a fatigued state. Spragg et al. [8] introduced a protocol consisting of a 20-minute effort at 50–70% of critical power (CP), followed by five 8-minute intervals at 105–110% of CP. These protocols have primarily been tested in small samples of elite or professional cyclists and have not been broadly replicated. Critical power itself has been increasingly recognized as a relevant fatigue threshold in endurance sports [10, 11].

To the best of our knowledge, there is currently no clear consensus on how to assess durability in either laboratory or field settings. Existing fatigue or durability protocols vary widely in their design (e.g., duration, intensity, endpoint definitions). Moreover, many of these studies rely on small sample sizes (*n* = 9–12), use non-standardized metrics, or are constrained to tightly controlled lab environments, which limits their generalizability to real-world training and competition contexts [3, 6, 8, 9]. Unlike the licensed U23 or professional cyclists studied in previous research, our participants are competitive but non-professional athletes who do not hold professional licenses. This distinction allows us to examine durability in a population that is often underrepresented in performance physiology research.

The recent pandemic has shifted not only changed training environments but also fundamentally altered the athlete–coach relationship, with fewer opportunities for direct, in-person supervision [12]. As a result, traditional lab-based testing has become less feasible and the need for standardized field-based assessment protocols that athletes can perform independently has increased. These field-based assessment protocols still yield meaningful and reproducible data, which can help in decision-making outside of laboratory settings.

Most coaches work not only with professional athletes, but also with juniors, U19, and amateur cyclists [13]. However, many of the above-mentioned durability testing protocols, such as those involving high total workload accumulation (e.g., 40 kJ/kg or prolonged submaximal rides over 3–4 h), are neither practical nor appropriate for these groups due to their physiological capacity, training age, or time constraints. Therefore, there is a growing need for standardized and scalable protocols that are both, physiologically meaningful and feasible across a broader range of athlete populations —without the need for laboratory equipment or direct supervision.

So far, most existing durability research has focused exclusively on elite or professional athletes. Well-trained amateur cyclists represent a large and often overlooked segment of competitive endurance sport. The study aimed to determine whether commonly used metrics in endurance performance diagnostics can meaningfully predict durability in real-world field settings. Therefore, the objective of this study was to investigate the relationship between durability and traditional physiological parameters such as VO₂ max, training volume, years of training, and age in a group of well-trained amateur cyclists.

We hypothesized that durability would not significantly correlate with conventional physiological variables. This would suggest that durability is a distinct and independent component of endurance performance, even in well-trained athletes, and requires specific methods for accurate assessment.

## Materials and methods

### Participants

Twenty well-trained male cyclists took part in the present investigation (age 34,6 ± 7,3 years; body weight: 74,4 ± 8,7 kg; 3,4 ± 1,06 years in endurance sport; weekly training volume, 8,21 ± 2 h·week-1). Body weight was measured prior the test under fresh conditions. The VO₂ max data were obtained from the last standardized cardiopulmonary exercise test performed no longer than four months before the start of the study using the COSMED Quark (COSMED, Rome, Italy) or VO2Master Pro (VO2 Master Health Sensors, Vernon, British Columbia, Canada).

All participants were free of recent (< 4 months) infection and chronic disease and habitually training > 6 h·week^− 1^ in cycling-based endurance sports.

### Control test

Testing was conducted over two consecutive weekends. Participants were instructed to consume a standardized breakfast of ~ 2 g·kg − 1 carbohydrate and ~ 800 mL of water one hour prior to testing, following general pre-workout nutritional guidelines [14]. Subjects were instructed to abstain from caffeine, strenuous exercise, and alcohol 24 h before each test and to standardize their food intake the night before. During the two tests, carbohydrate intake was standardized to 60 g·h − 1, using a 1:1 ratio of glucose to fructose in the form of energy drinks and gels. Participants did not consume caffeine during the fatigue protocol and were allowed to drink water ad libitum. We don’t have any documented cases of suspected non-compliance or reported deviations from our instructions from athletes.

**Fig. 1:**
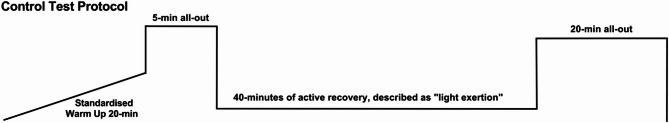
Control test protocol to estimate 5-minutes and 20-minutes all-out power output under fresh conditions

After a 20-minutes standardized warm-up phase, the first test was conducted in the form of 5-minutes and 20-minutes performance tests under fresh conditions (power output (PO)-5min_fresh_ and PO-20min_fresh_). The 5-minutes and 20-minutes all out test in fresh condition was separated by 40 minutes of active recovery, during which participants maintained a rating of perceived exertion (RPE) that did not exceed 2 out of 10 on Borg’s 10-point scale, equivalent to ‘light effort’ (Fig. 1) [15].

Before each effort in both, the fresh and the fatigue state, participants were asked to reach a maximum workload while maintaining a cadence between 80 and 100 revolutions per minute (rpm). The power values of the 5-minutes and 20-minutes all-out effort were plotted against 1/time (t), with t as the corresponding duration, to linearize the relationship between power and duration.

### Fatigue protocol

**Fig. 2:**
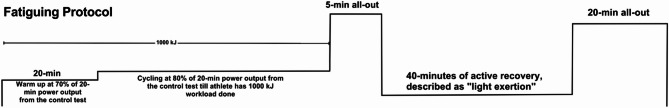
Fatigue test protocol to estimate 5-minutes and 20-minutes all-out power output under fatigue conditions

Prior to the second set of 5-minutes and 20-minutes tests, participants completed a fatigue protocol consisting of:

- 20 min at 70% of their initial PO-20min_fresh_ as a warm-up.
- Continuous cycling at 80% of the initial PO-20min_fresh_ until they had expended 1000 kJ of work. The total workload was calculated together with the first 20-minutes warm-up phase.
- Immediately afterwards, a 5-minutes all-out test was performed. (PO-5min_fatigue_).
- 5-minutes all-out effort was followed by 40-minutes of active recovery in which participants were instructed to not exceed an RPE of 2 out of 10 on the Borg 10-point scale (corresponding to the verbal anchor of “light exertion”) before proceeding to the subsequent effort. During the active recovery participants were allowed to freely choose their cadence.
- A 20-minutes all-out test is carried out immediately afterwards. (PO-20min_fatigue_) (Fig. 2).

### Power data analysis

All tests were performed with the participants’ own racing bikes on direct-drive, electromagnetically braked indoor cycling trainers (Kickr v5, Wahoo Fitness, Atlanta, USA; Tacx, Tacx, Wassenaar, Netherlands). These indoor trainers were calibrated according to the manufacturers’ specifications to ensure valid and reliable performance measurements. All participants used fans for cooling and performed the tests in a well-ventilated room. Air temperature ranged between 15 °C and 20 °C. Performance was measured using power meters from two brands: Garmin Rally (Garmin Ltd., Olathe, Kansas, USA) and Assioma (Favero Electronics, Treviso, Italy), recorded on wearable head-mounted devices (Garmin Edge 520 or Garmin Edge 1040, Garmin Ltd., Olathe, Kansas, USA). Participants performed a zero-point calibration before each test according to the manufacturer’s guidelines. Data files with irregularities, such as missing or erroneous power measurements or incomplete data due to technical problems (e.g. battery failure), were excluded from the analysis. The power data, i.e. the amount of work performed in kJ, was analyzed and visually inspected for errors by two independent researchers using commercially available cycling software (WKO 5 build 590, Peaksware LLC, Lafayette, CO, USA).

### Statistical analysis

All data were tested for normality using the Shapiro-Wilk test. For descriptive purposes, all data are expressed as mean ± standard deviation (mean ± SD) or mean difference (MD). Changes in output power were expressed as a percentage. The percentage changes were transformed into logits to fulfill the requirements of the multiple regression analysis approach [16].

A power analysis and a sensitivity analysis were conducted using G*Power to determine the required sample size and effect size of 0.35 for meaningful results. Assuming a large effect size, the power analysis indicated a minimum sample size of 20 participants. Additionally, the sensitivity analysis revealed that a minimum R^2^ of 25%. The assumption of a strong effect of the independent variables training, VO2max, years in sport, and age is based on the results of literature research in the context of sports [1, 3, 4, 17].

In addition, extensive regression diagnostics were carried out. This included assessments of multicollinearity (VIF), influential cases (Cook’s distance and leverage values), homoscedasticity (Goldfeld–Quandt test), linearity (RESET test), absence of autocorrelation (Durbin–Watson test), and normal distribution of residuals (Shapiro–Wilk test on residuals). Pairwise comparisons are made using the paired t-test. Statistical significance was set at *p* ≤ 0.05. The CW1 research tool (CW1 AB; Torggatan 8; 41105 Gothenburg SWE) was used to anonymize the data to ensure participant confidentiality throughout the study. Statistical analyses were performed using R (The R Foundation, Vienna, Austria). All figures and diagrams were also created using R. Specifically, we used the following packages: *tidyverse* (for data manipulation and visualization), mosaic (for basic statistics), *lmtest* (for diagnostic checking in linear regression models), and *ggplot2* (for plotting).

## Results

### Individual power output values

**Fig. 3:**
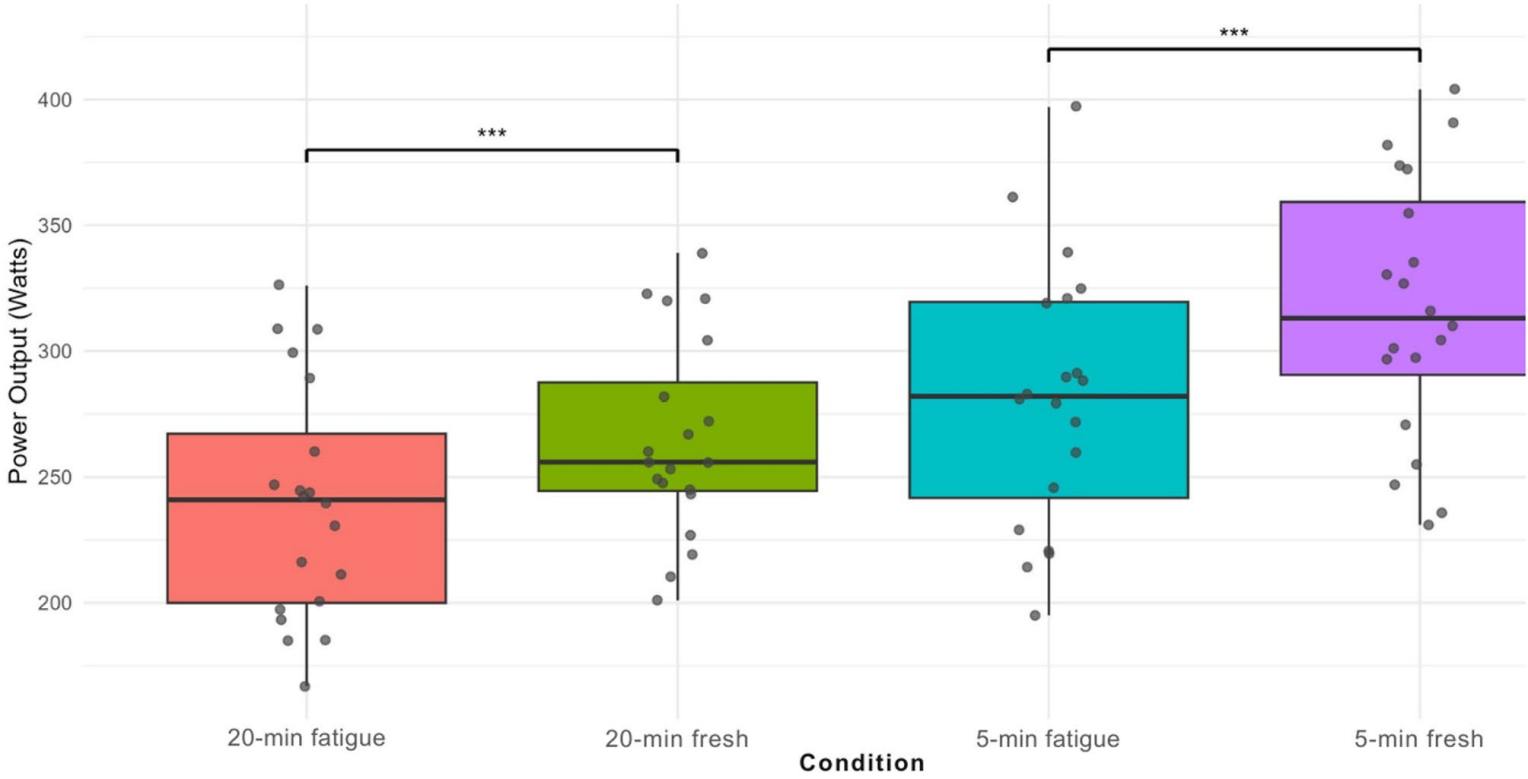
Comparison of 20-minutes and 5-minutes power output in fresh and fatigue conditions (***p < 0.01)

All data were normally distributed. Participants’ performance characteristics are listed in Table 1 (mean ± SD). Figure 3 presents a comparison of power output under fresh and fatigued conditions.

**Table 1:** Participants’ descriptive performance characteristics

PO-5min_fresh_ (W⋅kg^− 1^)^a^	PO-5min_fatigue_ (W⋅kg^− 1^)	PO-20min_fresh_ (W⋅kg^− 1^)	PO-20min_fatigue_ (W⋅kg^− 1^)	Drop of power in 5 min test (%)	Drop of power in 20 min test (%)
4.3 ± 0.8	3.8 ± 0.9	3.5 ± 0.6	3.2 ± 0.8	10.8 ± 6.5	10.1 ± 7.8

^*a*^Data are present as mean ± SD

PO-5min_fresh_ and PO-20min_fresh_ average relative power output in fresh condition, PO-5min_fatigue_ and PO-20min_fatigue_ average relative power output in fatigued condition

PO-20min_fresh_ and PO-20min_fatigue_ were significantly different (MD = 24,9 W, *P* < 0.001) as were PO-5min_fresh_ and PO-5min_fatigue_ (MD = 35,2, *P* < 0.001)

### Relationship between traditional parameters of physiology and durability

Multiple regression is used to investigate the influence of covariates on durability because it allows for the simultaneous examination of multiple predictors and their unique contributions to the dependent variable durability. By controlling other factors, this approach isolates the specific effect of each covariate, providing a clearer understanding of its role. Table 2 presents the results of two multiple regression models. The values in each cell represent the estimated regression coefficients from the respective model; standard errors are reported in parentheses.

**Table 2:** Correlations between drop of power for 5 min and 20 min and traditional physiological and training parameters

	Dependent variable:	
	Logit(5 min drop)	Logit(20 min drop)
	(1)	(2)
**Training volume**	0.0002	-0.001
	(0.002)	(0.002)
**VO2 max**	-0.028	-0.053
	(0.042)	(0.031)
**Years in sport**	0.089	0.122
	(0.239)	(0.178)
**Age**	0.022	-0.005
	(0.038)	(0.028)
**Constant**	-2.002	0.629
	(2.668)	(1.985)
**Observations**	20	20
R^2^	0.127	0.392
Adjusted R^2^	-0.105	0.230
**Residual Std. Error (df = 15)**	0.911	0.678
**F Statistic (df = 4; 15)**	0.547 (*p* = 0.704)	2.418 (*p* = 0.095)

Note: ^*^p < 0.05; ^**^p < 0.01; ^***^p < 0.001

Standard errors in parentheses

The model diagnosis shows the presence of multicollinearity due to the highly correlated variables VO_2_ max and W/kg (*r* = 0.95). Therefore, W/kg is removed from the regression models. After this correction, no unusual observations can be detected using Cook’s distance and leverage values. Likewise, homoscedasticity (Goldfeld-Quant Test: *p* > 0.05), linearity (RESET test: *p* > 0.05), no autocorrelation (Durbin-Watson test: *p* > 0.05) and normal distribution of the residuals (Shapiro-Wilk test: *p* > 0.05) are shown. Accordingly, the estimation of the regression coefficients is unbiased and the testing of the regression coefficients is reliable.

Models 1 and 2 show no significant F-tests (model 1: *p* = 0.704, model 2: *p* = 0.095). That indicates that the models, as a whole, does not explain a significant proportion of the variance in the dependent variable, suggesting that the predictors may not collectively contribute meaningfully to the outcome. The available covariates thus show no correlation with durability, even though important influencing variables such as VO_2_ max and training volume were used as covariates.

## Discussion

Durability in endurance sports refers to an athlete’s ability to maintain a high level of performance over extended periods of time, particularly under conditions of accumulated fatigue. The main aim of this study was to show that durability is a factor in cycling performance independent of traditional performance metrics.

While FTP and VO₂ max are well-established indicators of an athlete’s aerobic capacity and performance potential, emerging evidence suggests that durability is not directly correlated with these traditional metrics [4, 17]. This discrepancy highlights the complexity of endurance performance and the need to consider additional physiological and biomechanical factors. The concept of what we now call durability has been known for decades and is described in various forms [18]. It has been proposed by scientists that success of athletes in competitive sports is not solely dependent on traditional physiological parameters, such as maximal oxygen uptake or FTP [19]. Burnley and Jones discussed the critical power concept, indicating that endurance performance below the lactate threshold is influenced by factors beyond maximal aerobic [20]. Later in 2009, it was shown that mitochondrial biogenesis and oxidative enzyme activities are crucial for endurance performance, independent of VO₂ max [21]. Coates and colleagues found that long-distance runners with similar VO₂ max values had different performances in ultra-endurance events, suggesting that other factors contribute to sustained performance [22]. An analysis of the marathon performance of Paula Radcliffe who herself held the Women’s World Marathon Record with a time of 2:15:25 for 16 years, showed that her exceptional running economy and ability to sustain a high percentage of her VO₂ max were more influential on her performance than her VO₂ max itself [23]. In a review article, Bosquet [24] has discussed the limitations of VO₂ max as a sole predictor of endurance performance, emphasizing the importance of other factors such as mechanical efficiency and lactate threshold.

Economy of movement can potentially influence durability. In another review article, Joyner and Coyle discussed how factors such as a lactate threshold and exercise economy are critical determinants of endurance performance [5]. The article emphasizes that while VO₂ max sets the upper limit for aerobic metabolism, other factors like muscle efficiency and metabolic adaptations play significant roles [5]. The paper of Saunders emphasizes that there is considerable variation in running economy among runners and that economy is a robust predictor of performance [25]. This variation is independent of VO₂ max and lactate threshold, suggesting that mechanical and neuromuscular factors contribute significantly to endurance performance. Another study by Lucia and colleagues found that Eritrean elite runners had a superior running economy and lower energy cost of running compared to other elite runners [26]. Their VO₂ max values were not significantly higher, indicating that running economy was a key factor in their performance, which can be a predisposition for better durability. Another observation by Santalla and colleagues demonstrated, that elite cyclists improved their muscle efficiency over several years of training without significant changes in VO₂ max [27]. This improvement correlated with a better endurance performance, emphasizing the role of efficiency in long-term athletic development.

When considering possible differences in cycling success, the relationship between endurance and central regulation of fatigue could also be important. Tim Noakes suggested [28], that fatigue in endurance sport is regulated by the central nervous system, naming it “the central governor model” [29]. Noakes proposed that the brain regulates endurance performance through perceived effort to prevent physiological harm. Mental resilience allows endurance athletes to modulate this perception, pushing boundaries while maintaining safety. This process takes place in the brain outside of conscious control. Furthermore, changes in homeostasis of sarcoplasmic reticulum of muscles have been described and termed peripheral fatigue [30].

MacNamara and colleagues explored the psychological characteristics of elite athletes, including cyclists [31]. They found that mental toughness, comprised of confidence, focus, and resilience, was a distinguishing factor between elite and sub-elite performers. In cycling, resilience enables riders to cope with physical discomfort, tactical challenges, and unexpected events during races [32]. Elite cyclists demonstrated superior ability to handle pressure, recover from setbacks, and maintain a winning mindset [33].

Finally, also genetic predisposition needs to be taken into consideration. International elite runners and cyclists are more likely to have certain genetic variants compared to the less successful athletes. In a recent meta-analysis it was confirmed that international elite endurance athletes have a unique genetic make-up that is likely to contribute to their level of performance [34]. However, only a few genes are consistently associated with elite athletic performance, and none are strong enough to justify their use in predicting ability to perform at highest level [35]. Although a genetic predisposition cannot be discounted, reports indicate that experienced older cyclists with extensive training histories demonstrate remarkable resilience. This suggests that age and consistent, long-term, potentially extensive training may be significant factors that contribute to resilience [36].

It would be beneficial to consider whether the forenamed factors, such as VO_2_ max, lactate threshold, movement efficiency, the mental set of an athlete, and peripheral and central fatigue, could be identified as predictors of durability. It would be inaccurate to claim that a comprehensive range of predictors of durability has been identified, as only a limited number of studies have investigated this topic. Furthermore, our findings are consistent with previous research that found no associations between time trial performance decrements following prolonged cycling training and traditional parameters assessed in fresh conditions (e.g. VO_2_ max, FTP) [6, 37]. Consequently, it is impossible to make assumptions about an athlete’s level of durability based on physiological performance or performance at rest. In order to obtain a comprehensive physical assessment, durability must also be measured.

Spragg and colleagues identified robust correlations between durability (i.e. relative reductions in critical power) and laboratory-based measures obtained in a fresh state (e.g. VO_2_ max, second ventilatory threshold) [8]. These findings were not evident in our study. Consequently, some of the observed differences between the various studies are likely to be due to non-standardized test protocols, highlighting the need to define a reproducible and reliable fatigue endurance protocol. Differences in fatigue protocols are likely to affect the contribution of mechanisms underlying fatigue and endurance (e.g. glycogen utilization, fiber contractile properties and central factors), and hence laboratory-based predictors of durability [38]– [39]. Studies like those by Hvid et al. [38] and Jensen et al. [40] highlight that muscle fiber contractile properties, Ca²⁺ sensitivity, and intramyofibrillar glycogen depletion play crucial roles in fatigue resistance. Passfield et al. [37] further linked post-fatigue performance declines to decreased gross efficiency. Together, these findings underscore that durability is multifactorial and requires integrated testing approaches that account for metabolic, neuromuscular, and central contributions.

Durability can be assessed in both laboratory and field settings. Hovever, each approach has unique advantages and limitations. In laboratory protocols, cyclists typically perform a CP testing in fresh state and another CP testing after high-intensity workout (e.g., five bouts of 8-min of exercise at 105–110% of CP) to induce fatigue [8]. Spragg and colleagues showed that reductions in CP were strongly associated with multiple laboratory-based performance variables. These protocols reflect the concept of a dynamic CP, which declines with accumulated work, and may serve as a sensitive marker of durability.

In contrast, field-based assessments rely on power data collected from training or controlled fatigue protocol, such as multi-hour rides followed by maximal efforts, to observe performance declines under real-world conditions [9]. A notable field-based approach to assessing durability was introduced by van Erp et al. [7], who analysed maximal mean power outputs over different durations (20 min, 5 min, 1 min, and 10 s) relative to body weight across multiple seasons in professional cyclists. To examine how prior work affects performance, they categorized efforts based on accumulated mechanical work, expressed in kilojoules per kilogram (kJ·kg⁻¹) with six different thresholds: 0, 10, 20, 30, 40, and 50 kJ·kg⁻¹. This method allowed for the investigation of fatigue-induced declines in performance under real-world conditions and highlighted how sustained submaximal workload impacts the capacity to produce peak efforts. In elite cycling, durability manifests as the ability to sustain race-deciding attacks or climbs in the final stages of long races. General classification contenders are capable to produce near-threshold or supra-threshold power late in multi-hour efforts, highlighting the competitive significance of fatigue resistance. Our findings emphasize that such performance capacity is not adequately predicted by traditional markers alone and must be assessed independently.

For many athletes and coaches, durability is not a new concept but rather a formalization of observations they have made over years of training and competition. These individuals have noticed for a long time that some athletes can maintain higher power outputs over extended periods even if they do not have the highest VO₂ max or FTP.For these practitioners, durability validates their experiences and provides a scientific framework to explain why some athletes excel in endurance events despite not topping the charts in VO₂ max or FTP tests.

However, some riders and coaches find the concept of durability novel and difficult to integrate into their existing training philosophies. For these individuals, the idea that durability operates independently of traditional physiological markers may seem counterintuitive. It takes a change of perspective to realize that an athlete’s ability to resist fatigue and maintain performance is a special characteristic that requires special attention.

In addition, previous research on durability and fatigue resistance has primarily focused on elite or professional adult cyclists. Our study extends this understanding to well-trained amateur athletes using a novel, field-based test and examining its relationship with standard physiological metrics. This has practical implications for training, performance monitoring, and talent identification, particularly in younger or non-professional athlete populations. By integrating durability into training practices, cyclists and coaches at all levels can work toward optimizing performance and redefining the boundaries of endurance capacity.

### Limitations

This study is limited by sample size. In our data, the model predicting correlation between 20-minute drop in power and physiological parameters (Model 2) achieved an R² of 0.23 (f² = 0.3) with a significant overall F-test for the regression at a 10% significance level, which makes the regression results usable, but, indicating very scarce insufficient power. In contrast, model 1 shows no correlations at all. We evaluate these results as sufficient to conclude that durability is not influenced by the variables we measured. Nevertheless, this should also occur in future with larger samples.

The effects of durability on endurance performance should be investigated in a wider range of athlete populations, and sports to provide a more detailed understanding of how durability influences real-world endurance performance outcomes. Furthermore, external factors such as diet, fluid intake, sleep, quality of life, stress level and mental resilience were not systematically controlled, although they probably influence endurance. The lack of detailed metabolic and biochemical analyses, such as mitochondrial function or oxidative stress markers, also limits our understanding of the underlying mechanisms. Future studies should address these gaps to provide a more comprehensive picture of durability and its role in endurance performance. Another limitation of the study is that all of the data were collected from male athletes. It remains unclear whether a similarly strong relationship exists between durability and race performance in female cyclists.

### Practical implications and conclusions

Fatigue resistance does not appear to be dependent on traditional parameters of endurance performance and needs to be investigated from a different and more complex perspective. Exercise physiologists, scientists and coaches need to come together to create a single standardized protocol for measuring durability in cycling, because valid, and standardized performance tests are essential for generating accurate, consistent, and actionable results that can guide training and performance strategies [41]. In order to sufficiently substantiate the model presented here in the future, further scientific work with larger samples is absolutely necessary.

## Data Availability

Data are available on reasonable request to the corresponding author.

## Abbreviations

CP: Critical power
VO_2_ max: Maximal oxygen uptake
PO: Power output
RPE: Rating of perceived exertion
rpm: Revolutions per minute rpm
SD: Standard deviation
MD: Mean difference
FTP: Functional threshold power

## References

[1] van Erp T, Sanders D, de Koning JJ. Training characteristics of male and female professional road cyclists: a 4-year retrospective analysis. Int J Sports Physiol Perform. 2019;15(4):534–40. 10.1123/ijspp.2019-0320. 31722298 10.1123/ijspp.2019-0320

[2] Mujika I, Padilla S. Physiological and performance characteristics of male professional road cyclists. Sports Med. 2001;31(7):479–87. 10.2165/00007256-200131070-00003. 11428685 10.2165/00007256-200131070-00003

[3] Leo P, Spragg J, Mujika I, Giorgi A, Lorang D, Simon D, et al. Power profiling, workload characteristics, and race performance of U23 and professional cyclists during the multistage race tour of the alps. Int J Sports Physiol Perform. 2021;16(8):1089–95. 10.1123/ijspp.2020-0381. 33789246 10.1123/ijspp.2020-0381

[4] Maunder E, Seiler S, Mildenhall MJ, Kilding AE, Plews DJ. The importance of durability in the physiological profiling of endurance athletes. Sports Med. 2021;51(8):1619–28. 10.1007/s40279-021-01459-0. 33886100 10.1007/s40279-021-01459-0

[5] Joyner MJ, Coyle EF. Endurance exercise performance: the physiology of champions. J Physiol. 2008;586(1):35–44. 10.1113/jphysiol.2007.143834. 17901124 10.1113/jphysiol.2007.143834 PMC2375555

[6] Valenzuela PL, Alejo LB, Ozcoidi LM, Lucia A, Santalla A, Barranco-Gil D. Durability in professional cyclists: a field study. Int J Sports Physiol Perform. 2023;18(1):99–103. 10.1123/ijspp.2022-0202. 36521188 10.1123/ijspp.2022-0202

[7] van Erp T, Sanders D, Lamberts RP. Maintaining power output with accumulating levels of work done is a key determinant for success in professional cycling. Med Sci Sports Exerc. 2021;53(9):1903–10. 10.1249/MSS.0000000000002656. 33731651 10.1249/MSS.0000000000002656

[8] Spragg J, Leo P, Swart J. The relationship between physiological characteristics and durability in male professional cyclists. Med Sci Sports Exerc. 2023;55(1):133–40. 10.1249/MSS.0000000000003024. 35977108 10.1249/MSS.0000000000003024

[9] Leo P, Giorgi A, Spragg J, Gonzalez BM, Mujika I. Impact of prior accumulated work and intensity on power output in elite/international level road cyclists—a pilot study. Ger J Exerc Sport Res. 2022;52(4):673–7. 10.1007/s12662-022-00818-x.

[10] Poole DC, Burnley M, Vanhatalo A, Rossiter HB, Jones AM. Critical power: an important fatigue threshold in exercise physiology. Med Sci Sports Exerc. 2016;48(11):2320–34. 10.1249/mss.0000000000000939. 27031742 10.1249/MSS.0000000000000939 PMC5070974

[11] Saif A, Khan Z, Parveen A. Critical power as a fatigue threshold in sports: A scoping review. Sci Sports. 2022;37(8):703–9. 10.1016/j.scispo.2021.05.010.

[12] Kirkland A, Cowley J. An exploration of context and learning in endurance sports coaching. Front Sports Act Living. 2023;5:1147475. 10.3389/fspor.2023.1147475. 37139300 10.3389/fspor.2023.1147475 PMC10150095

[13] Leo P, Spragg J, Wakefield J, Swart J. Predictors of cycling performance success: traditional approaches and a novel method to assess performance capacity in U23 road cyclists. J Sci Med Sport. 2023;26(1):52–7. 10.1016/j.jsams.2022.11.005. 36513568 10.1016/j.jsams.2022.11.005

[14] Podlogar T, Wallis GA. New horizons in carbohydrate research and application for endurance athletes. Sports Med. 2022;52(Suppl 1):5–23. 10.1007/s40279-022-01757-1. 36173597 10.1007/s40279-022-01757-1 PMC9734239

[15] Borg GA. Psychophysical bases of perceived exertion. Med Sci Sports Exerc. 1982;14(5):377–81. 10.1249/00005768-198205000-00012. 7154893

[16] Seiffert S, Weber S, Sack U, Keller T. Use of logit transformation within statistical analyses of experimental results obtained as proportions: example of method validation experiments and EQA in flow cytometry. Front Mol Biosci. 2024;11:11:1335174. 10.3389/fmolb.2024.1335174. 39055985 10.3389/fmolb.2024.1335174 PMC11269570

[17] Sørensen A, Aune TK, Rangul V, Dalen T. The validity of functional threshold power and maximal oxygen uptake for cycling performance in moderately trained cyclists. Sports. 2019;7(10):217. 10.3390/sports7100217. 31581544 10.3390/sports7100217 PMC6835290

[18] Noakes TD. Physiological models to understand exercise fatigue and the adaptations that predict or enhance athletic performance. Scand J Med Sci Sports. 2000;10(3):123–45. 10.1034/j.1600-0838.2000.010003123.x. 10843507 10.1034/j.1600-0838.2000.010003123.x

[19] Thomas K, Goodall S, Stone M, Howatson G, St Clair Gibson A, Ansley L. Central and peripheral fatigue in male cyclists after 4-, 20-, and 40-km time trials. Med Sci Sports Exerc. 2015;47(3):537–46. 10.1249/MSS.0000000000000448. 25051388 10.1249/MSS.0000000000000448

[20] Burnley M, Jones AM. Oxygen uptake kinetics as a determinant of sports performance. Eur J Sport Sci. 2007;7(2):63–79. 10.1080/17461390701456148.

[21] Marcuello A, Martínez-Redondo D, Dahmani Y, Terreros JL, Aragonés T, Casajús JA, et al. Steady exercise removes vo₂max difference between mitochondrial genomic variants. Mitochondrion. 2009;9(5):326–30. 10.1016/j.mito.2009.04.007. 19427920 10.1016/j.mito.2009.04.007

[22] Coates AM, Berard JA, King TJ, Burr JF. Physiological determinants of ultramarathon trail-running performance. Int J Sports Physiol Perform. 2021;16(10):1454–61. 10.1123/ijspp.2020-0766. 33691287 10.1123/ijspp.2020-0766

[23] Jones AM. The physiology of the world record holder for the women’s marathon. Int J Sports Sci Coach. 2006;1(2):101–16. 10.1260/174795406777641258.

[24] Bosquet L, Léger L, Legros P. Methods to determine aerobic endurance. Sports Med. 2002;32(11):675–700. 10.2165/00007256-200232110-00002. 12196030 10.2165/00007256-200232110-00002

[25] Saunders PU, Pyne DB, Telford RD, Hawley JA. Factors affecting running economy in trained distance runners. Sports Med. 2004;34(7):465–85. 10.2165/00007256-200434070-00005. 15233599 10.2165/00007256-200434070-00005

[26] Lucia A, Esteve-Lanao J, Oliván J, Gómez-Gallego F, San Juan AF, Santiago C, et al. Physiological characteristics of the best Eritrean runners—exceptional running economy. Appl Physiol Nutr Metab. 2006;31(5):530–40. 10.1139/h06-029. 17111007 10.1139/h06-029

[27] Santalla A, Naranjo J, Terrados N. Muscle efficiency improves over time in world-class cyclists. Med Sci Sports Exerc. 2009;41(5):1096–101. 10.1249/MSS.0b013e318191c802. 19346977 10.1249/MSS.0b013e318191c802

[28] Noakes TD. J.B. Wolffe memorial lecture. Challenging beliefs: ex Africa semper aliquid Novi. Med Sci Sports Exerc. 1997;29(5):571–90. 10.1097/00005768-199705000-00001. 9140893 10.1097/00005768-199705000-00001

[29] Noakes TD. Fatigue is a brain-derived emotion that regulates the exercise behavior to ensure the protection of whole body homeostasis. Front Physiol. 2012;3:82. 10.3389/fphys.2012.00082. 22514538 10.3389/fphys.2012.00082 PMC3323922

[30] Fitts RH. Cellular mechanisms of muscle fatigue. Physiol Rev. 1994;74(1):49–94. 10.1152/physrev.1994.74.1.49. 8295935 10.1152/physrev.1994.74.1.49

[31] MacNamara Á, Button A, Collins D. The role of psychological characteristics in facilitating the pathway to elite performance part 1: identifying mental skills and behaviors. Sport Psychol. 2010;24(1):52–73. 10.1123/tsp.24.1.52.

[32] Sarkar M, Fletcher D. Psychological resilience in sport performers: a review of stressors and protective factors. J Sports Sci. 2014;32(15):1419–34. 10.1080/02640414.2014.901551. 24716648 10.1080/02640414.2014.901551

[33] Galli N, Vealey RS. Bouncing back from adversity: athletes’ experiences of resilience. Sport Psychol. 2008;22(3):316–35. 10.1123/tsp.22.3.316.

[34] Konopka MJ, van den Bunder JCM, Rietjens G, Sperlich B, Zeegers MP. Genetics of long-distance runners and road cyclists—a systematic review with meta-analysis. Scand J Med Sci Sports. 2022;32(10):1414–29. 10.1111/sms.14212. 35839336 10.1111/sms.14212 PMC9544934

[35] Guth LM, Roth SM. Genetic influence on athletic performance. Curr Opin Pediatr. 2013;25(6):653–8. 10.1097/MOP.0b013e3283659087. 24240283 10.1097/MOP.0b013e3283659087 PMC3993978

[36] Almquist NW, Hansen J, Rønnestad BR. Development of cycling performance variables and durability in female and male National team cyclists: from junior to senior. Med Sci Sports Exerc. 2023;55(11):2053–63. 10.1249/MSS.0000000000003232. 37259247 10.1249/MSS.0000000000003232

[37] Passfield L, Doust JH. Changes in cycling efficiency and performance after endurance exercise. Med Sci Sports Exerc. 2000;32(11):1935–41. 10.1097/00005768-200011000-00018. 11079525 10.1097/00005768-200011000-00018

[38] Millet GY, Lepers R. Alterations of neuromuscular function after prolonged running, cycling and skiing exercises. Sports Med. 2004;34(2):105–16. 10.2165/00007256-200434020-00004. 14965189 10.2165/00007256-200434020-00004

[39] Hvid LG, Gejl K, Bech RD, Nygaard T, Jensen K, Frandsen U, et al. Transient impairments in single muscle fibre contractile function after prolonged cycling in elite endurance athletes. Acta Physiol. 2013;208(3):265–73. 10.1111/apha.12095. 10.1111/apha.12095 23480612

[40] Jensen R, Ørtenblad N, Stausholm MLH, Skjærbæk MC, Larsen DN, Hansen M, et al. Heterogeneity in subcellular muscle glycogen utilisation during exercise impacts endurance capacity in men. J Physiol. 2020;598(19):4271–92. 10.1113/JP280247. 32686845 10.1113/JP280247

[41] Currell K, Jeukendrup AE. Validity, reliability and sensitivity of measures of sporting performance. Sports Med. 2008;38(4):297–316. 10.2165/00007256-200838040-00003. 18348590 10.2165/00007256-200838040-00003

